# Successful Treatment of Hemorrhagic Bullous Henoch-Schönlein Purpura with Oral Corticosteroid: A Case Report

**DOI:** 10.1155/2013/680208

**Published:** 2013-04-16

**Authors:** Celebi Kocaoglu, Ramazan Ozturk, Yasar Unlu, Fatma Tuncez Akyurek, Sukru Arslan

**Affiliations:** ^1^Department of Pediatrics, Konya Education and Research Hospital, 42090 Konya, Turkey; ^2^Department of Pathology, Konya Education and Research Hospital, 42090 Konya, Turkey; ^3^Department of Dermatology, Konya Education and Research Hospital, 42090 Konya, Turkey; ^4^Department of Pediatric Rheumatology, Konya Education and Research Hospital, 42090 Konya, Turkey

## Abstract

Henoch-Schönlein purpura (HSP) is a vasculitis of small-sized blood vessels, resulting from immunoglobulin-A-mediated inflammation. It is the most common acute systemic vasculitis in childhood and mainly affects skin, gastrointestinal tract, joints, and kidneys. The characteristic rash of HSP consists of palpable purpuric lesions 2 to 10 mm in diameter concentrating in the buttocks and lower extremities. The occurrence of hemorrhagic bullae in children with HSP is rarely encountered. This report describes a 4.5-year-old female patient with HSP associated with hemorrhagic bullous lesions.

## 1. Introduction

Henoch-Schönlein purpura (HSP) is a vasculitis of small-sized blood vessels, resulting from immunoglobulin-A-mediated inflammation. It is the most common acute systemic vasculitis in childhood and mainly affects skin, gastrointestinal (GI) tract, joints, and kidney [1]. The dominant clinical features of HSP are cutaneous purpura (100%), arthritis (82%), abdominal pain (63%), GI bleeding (33%), and nephritis (40%) [2]. The purpura is typically encountered on the legs and buttocks but may also be seen on the arms, face, and trunk. Several patients present with predominantly petechial lesions, some with mainly purpuric lesions, and others with a mixture type of lesions [3]. Some patients exhibit target-like lesions that consist of a central punctate hemorrhage surrounded by circumferential regions with pallor and hemorrhage [4]. Histologically, cutaneous features of HSP are characterized by leukocytoclastic vasculitis of the dermal vessels and prominent IgA deposits in the vessel walls [5].

GI manifestations of HSP include abdominal pain, vomiting, diarrhea, paralytic ileus, melena, intussusception, and mesenteric ischemia or perforation [6]. Ankles and knees may be involved frequently, but arthritis on hands, elbows, and feet could also be seen. Arthritis is nonerosive and hence causes no permanent deformity [7]. Involvements of some organs and systems such as central nervous and lungs may be observed, but are much less common than those in skin, bowel, and kidneys [8].

In our report, treated with oral corticosteroid, a 4.5-year-old female patient with HSP, especially accompanied by hemorrhagic bullous skin lesions, was presented in light of the literature.

## 2. Case Presentation

A 4.5-year-old girl was referred to Konya Education and Research Hospital with the complaints of hemorrhagic bullous rashes, bilateral ankle pain, and severe abdominal pain. There was no history of reduced urine output, visible blood in the urine, or black stool. The case had taken no medication and had no history of upper respiratory tract infections or animal/insect bites for at least 1 month prior to the onset of rashes. 

**Figure 1:**
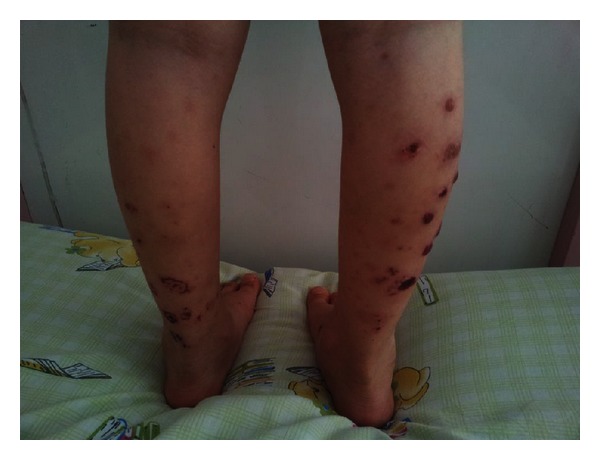
Palpable purpura and hemorrhagic bullae on both lower extremities.

On admission, vital findings were as follows: temperature, 36,3°C; heart rate, 90 bpm; respiratory rate, 26 breaths/min; and blood pressure, 85/60 mmHg. Numerous palpable purpura and hemorrhagic bullae, varying in size from 5 to 15 mm in diameter, were determined on her both lower extremities (Figure 1). The abdomen was soft but tender to deep palpation with hyperactive bowel sounds. No abdominal mass or hepatosplenomegaly was detected. The lower extremities showed no edema, but active and passive movements of both ankles were painful. Physical examination demonstrated no other abnormal findings.

Laboratory investigations revealed such rates of hemoglobin as 13,7 g/dL, of white blood cell count as 12.2 × 10^9^/L with a normal differential count, and of platelet count as 303 × 10^9^/L. A stool test for occult blood was positive. C-reactive protein and erythrocyte sedimentation rates were 33,9 mg/dL and 32 mm/h, respectively. Tests for ANA and c-ANCA were negative, and C3, C4, and serum immunoglobulin A were within normal limits. Serum total protein and albumin, transaminase, blood urea nitrogen, creatinine, and electrolytes were normal on admission. Urine dipstick test revealed ketone (4 plus), blood (2 plus), leukocyte (2 plus), and protein (2 plus). Two red blood cells and 4 leukocyte cells were determined at the urine sediment (high-powered microscopic field). The amounts of protein and urine were 23.4 mg/m^2^/h and 750 mL/day, respectively. On followup, the urine output was between 1.8 and 2 mL/kg/h. A stool test for occult blood was positive.

**Figure 2:**
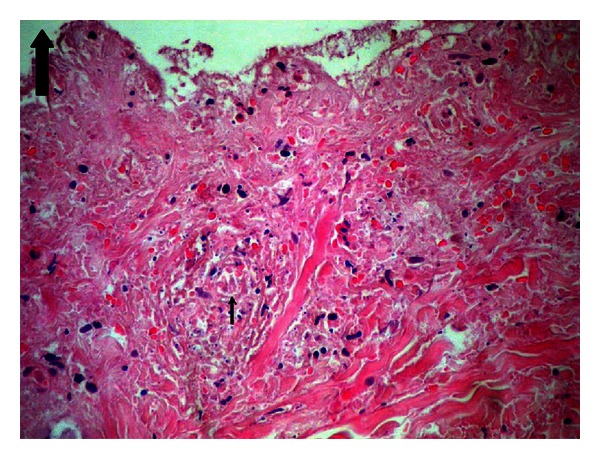
Neutrophilic inflammation with fibrinoid necrosis and fragmented neutrophilic nuclei (leukocytoclasis). HEX 300.

**Figure 3:**
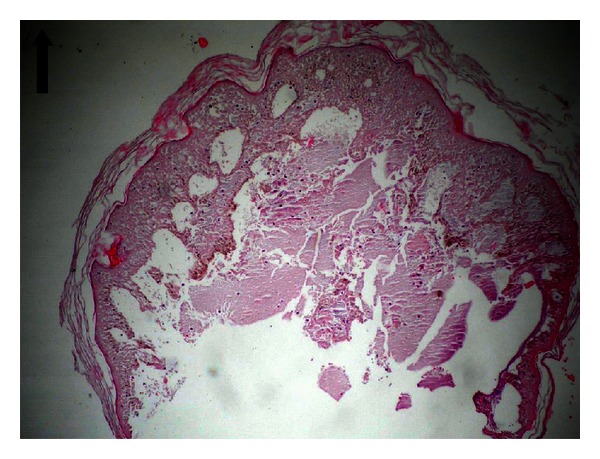
Development of subepidermal bullae and epidermal separation. HEX 100.

In light of clinical and laboratory findings, the case was diagnosed with HSP. As a result of biopsy performed with any of the lesions, the specimen was determined to show leukocytoclastic vasculitis with perivascular infiltration by polymorphonuclear leukocytes (Figures 2 and 3). Thus, the diagnosis of hemorrhagic bullous HSP was confirmed.

Because skin, joint, GI tract, and moderate renal involvements resulting from HSP were considered, the treatment of prednisolone at a dose of 2 mg/kg/day for 6 weeks and symptomatic treatment including bed rest along with a bland diet were started. The arthralgia and the abdominal pain improved 4 days after the initial therapy. The bullous lesions also began to resolve within a week and completely recovered in the course of 3-week treatment. Because 24-hour urine proteinuria was found to be 9.4 mg/m^2^/h following 4-week prednisolone treatment, the treatment of prednisolone was continued for additional 2 weeks. The treatment of prednisolone was discontinued gradually after decrease of 24-hour urine proteinuria to 3.54 mg/m^2^/h. The case was followedup for 4 months during which the urinalysis remained within normal limits, and no symptoms of HSP were observed.

## 3. Discussion

HSP is one of the most common vasculitis syndromes in childhood. Palpable purpura is commonly seen in almost 100% of patients with HSP and is considered to be the main reason of hospital visits in 50% of cases [4]. The most severe lesions are often noted under points of maximal pressure such as around the malleoli or on the dorsal aspect of the foot beneath tight shoelaces, and the bullae in our case are also consistent with the findings suggested in the study by Leung and Robson [9].

The pathophysiology of hemorrhagic bullous HSP still remains unclear. In a study by Kobayashi et al., matrix metalloproteinase-9 (MMP-9, gelatinase B) was reported to be elevated in the blister fluid using zymography, and MMP-9, secreted by PMN on the dermal side of the dermoepidermal junction, was postulated to migrate from the lesion of intensive vasculitis and to cause blister formation by degrading basement membrane components such as type VII collagen [10].

The occurrence of hemorrhagic bullae is rarely encountered in children with HSP. First time in 1985, Garland and Chusid, alerting other clinicians to be aware of atypical presentation of HSP, reported a 5-year-old boy with HSP who developed hemorrhagic bullae on the extensor surfaces of his elbows, thighs, buttocks, and perioral region [11]. In another study performed by Liu et al., such lesions in two cases were reported to become regressed with the administration of steroids and to show no relapses during followups [12]. In 2005, Ishii et al. reported a 4-year-old boy with HSP who developed haemorrhagic bullae, arthralgia, and severe abdominal pain unresponsive to conventional therapy and controlled with pulse steroid [13]. In a study performed by Trapani et al. in 2010, three cases with severe purpuric palpable rashes were reported. While no treatment was administered to the second case, the first and third cases were treated with oral prednisolone for three days, as well as pulse methylprednisolone. Due to the exacerbation of the condition, the administration of prednisolone had to be lengthened. In the third case, however, azathioprine was added to the treatment regime because of unsuccessful treatment of steroids [1]. As consistent with the report by den Boer et al., the amelioration period of bullous lesions was nearly three weeks in our case, and we considered that the shorter amelioration period was due to the use of prednisolone [14].

Furthermore, consensus may be suggested not to be present, regarding the use of corticosteroids in the management of HSP progressing with only skin lesions. However, our case displayed such findings as abdominal pain and arthralgia among significant symptoms of HSP, as well as the bullae, and well responded to the treatment with oral prednisolone. As well as moderate renal involvement, our case also indicated severe GI manifestations like abdominal pain and bleeding. Hence, prednisolone treatment was started, and symptoms like arthralgia and abdominal pain were seen to alleviate four days after the initial therapy.

Although no consensus exists in the management of extensive bullous lesions in HSP, the anti-inflammatory effect of corticosteroids is likely to be beneficial in the treatment of HSP patients with severe cutaneous involvement through inhibition of AP-1 binding activity in the nucleus in association with a reduction of nuclear factor-kappa B (NF-*κ*B), one of the major proinflammatory transcription factors, and decreased MMP-2 and MMP-9 concentrations in plasma levels [15].

As a conclusion, hemorrhagic bullae are rarely seen in children with HSP. In the assessment of children with hemorrhagic bullae, healthcare professionals should be vigilant for HSP accompanied by atypical skin lesions. Hemorrhagic bullous HSP, although controversial, is a well-responsive condition to conventional oral prednisolone therapy. Considering the possible side effects of corticosteroids and that some cases ameliorate spontaneously, especially in cases with no GI tract and renal involvements, whether corticosteroid treatment will be started or not should be decided by clinicians. In addition, further studies are needed to enlighten the effects of steroids in the management of cutaneous manifestations in HSP.
